# Effects of the difference foot strike pattern on the plantar pressure during uphill and downhill running

**DOI:** 10.3389/fspor.2025.1654489

**Published:** 2025-10-23

**Authors:** Yoshiki Horiguchi, Hiroaki Noro, Keiichiro Hata, Yohei Yamazaki, Atsushi Kubota, Toshio Yanagiya

**Affiliations:** ^1^ Graduate School of Health and Sports Science, Juntendo University, Chiba, Japan; ^2^ Achilles Corporation, Tokyo, Japan; ^3^ Institute of Sports Sciences & Medicine, Juntendo University, Chiba, Japan

**Keywords:** running, foot strike pattern, uphill and downhill, inclination, plantar pressure

## Abstract

The purpose of this study was to clarify the effects of foot strike patterns on plantar pressure distribution during uphill, level, and downhill running. Eleven participants performed six randomized trials on a treadmill running at 3.33 m/s, combining two foot strike patterns, rearfoot strike and forefoot strike with three slope conditions of −6°, 0° and +6°. Plantar pressure data were collected using a pressure measurement insole. Peak pressure, peak force, time to peak, and loading rate were calculated for the heel, midfoot, and forefoot regions. As a result, at the heel region, peak pressure in rearfoot strike increased by approximately 32.1% during downhill running and decreased by approximately 24.3% during uphill running compared to level running. At the forefoot region, regardless of the foot strike pattern, peak pressure decreased by approximately 6.8% during downhill running compared to level running, but no significant differences were observed between uphill and level running. These results provide a useful basis for the development of injury prevention and performance improvement during slope running.

## 1 Introduction

The foot strike pattern during running has been recognized as an important internal factor that influences the ground reaction force acting on the body. The foot strike patterns are generally classified into rearfoot strike and forefoot strike, both of which affect the characteristics of ground reaction forces and the risk of running related injuries. For instance, rearfoot strike tends to produce a distinct impact peak and a rapid increase in vertical ground reaction force known as the loading rate, whereas forefoot strike is associated with a substantial reduction of these components (1). Among these impact-related variables, a high loading rate has been linked to an increased risk of running related injuries, such as tibial stress fractures (2), and forefoot strike has been suggested to potentially reduce this risk (3). In addition, forefoot strike may enhance stiffness, an indicator of spring like behavior during running, potentially contributing to performance improvement (4).

On the other hand, undulation is a representative external factor that affects ground reaction force. In road races, runners often encounter uneven terrain, including uphill and downhill slopes (5), which can alter running mechanics and performance. For example, running speed changes depending on the angle of the slope (6), and downhill running increases the impact peak, loading rate, and braking force (7). In response, runners adapt their movement patterns by increasing hip and knee flexion (8–10). Therefore, runners are required to adjust their running kinematics and force output differently from level running. Moreover, changes in ground reaction forces due to slope may interact in complex ways with foot strike patterns.

Recent studies have clarified the interactive effects of foot strike pattern and slope on the generation of ground reaction forces and running biomechanics. In particular, it has been suggested that vertical ground reaction forces, such as the active peak (7), and anterior-posterior ground reaction forces, such as the propulsive and braking peaks, are influenced by both foot strike pattern and slope (11). In addition, the distribution and location of forces on the plantar surface, the point of application of ground reaction forces, may also vary depending on foot strike pattern and slope. High pressure applied to the forefoot region has been associated with tibial stress fractures (12), highlighting the importance of evaluating plantar pressure for injury prevention. However, the mechanical effects of different foot strike patterns on the plantar surface during slope running remain insufficiently understood.

Therefore, the aim of this study was to investigate the effects of foot strike pattern on plantar pressure during slope running. Two hypotheses were proposed: (1) During rearfoot strike, peak pressure, peak force, and loading rate at the heel region would increase during downhill running and decrease during uphill running compared to level running; and (2) forefoot peak pressure would increase during uphill running and decrease during downhill running regardless of foot strike pattern. The findings of this study are expected to contribute to the selection of appropriate foot strike patterns and the development of effective running strategies for slope running, as well as to inform injury prevention.

## 2 Methods

### 2.1 Participants

Eleven healthy male participants (mean age: 22.2 ± 1.27 years, height: 1.68 ± 0.05 m, body mass: 62.6 ± 4.84 kg) voluntarily took part in this study. The required sample size for this study was calculated using G*Power (version 3.1.9.7, University Kiel, Germany) (13). Based on an assumed effect size f = 0.35, a significance level of *α* = 0.05, and a statistical power of 0.80, the minimum required number of participants was estimated to be 10. The assumed effect size f = 0.35 was derived from the vertical impact loading rate data reported by Kowalski et al. (11) for slope conditions comparable to our study (−6°, 0°, and +6°). Means and standard deviations from their Table 1 were converted to Cohen's f, and the largest between-slope comparison (−6° vs. +6°) yielded f ≈ 0.35, which was used for the sample size calculation.

**Table 2:** Variables measured by the pressure sensing insole for each regions during running at each examined conditions. Values represent means for all 11 subjects ± standard deviation.

Characteristics[unit]	Regions	Downhill (−6°)		Level (0°)		Uphill (+6°)		Main Effect of Slope	Main Effect of FSP	Interaction	Post hoc	
		Rearfoot Strike	Forefoot Strike	Rearfoot Strike	Forefoot Strike	Rearfoot Strike	Forefoot Strike				Slope	FSP
Step Frequency (step/s)	-	3.1 ± 0.19	3.1 ± 0.32	3.1 ± 0.17	3.1 ± 0.18	3.3 ± 0.18	3.3 ± 0.20	**	n.s.	n.s.	DR, LR < UR	
Step Length (m)	-	1.08 ± 0.065	1.08 ± 0.109	1.08 ± 0.063	1.07 ± 0.063	1.02 ± 0.056	1.01 ± 0.063	*	n.s.	n.s.	DR > UR	
Contact Time (ms)	Heel	153.2 ± 15.76	121.5 ± 24.17	159.0 ± 23.62	101.6 ± 19.88	173.3 ± 19.45	80.1 ± 28.71	n.s.	**	**	RFS: DR < UR., FFS: n.s.	
	Midfoot	176.9 ± 23.69	148.7 ± 22.55	179.6 ± 29.18	157.6 ± 18.11	191.4 ± 20.87	170.0 ± 15.20	**	**	n.s	DR < UR	RFS > FFS
	Forefoot	234.2 ± 13.18	233.5 ± 14.09	227.8 ± 18.79	234.9 ± 16.50	229.5 ± 14.74	237.8 ± 14.42	n.s.	n.s.	n.s.	-	
	Entire	242.3 ± 12.48	232.5 ± 13.49	242.2 ± 15.81	233.6 ± 15.46	247.6 ± 12.48	236.5 ± 12.33	n.s.	**	n.s.	-	RFS > FFS

Results of post-tests indicate significant differences. The significance level is *α*  = 0.05.

DR, downhill running; LR, level running; UR, uphill running; FSP, foot strike pattern; RFS, rearfoot strike; FFS, forefoot strike.

* denotes a *p*-value less than 0.05.

** indicates a *p*-value of less than 0.001.

Participants were recruited from a single university and were eligible for inclusion if they met the following criteria: (1) shoe size of 26.0 cm (US 8.0), and (2) no history of lower limb injury in the past six months. The criterion regarding shoe size was set to standardize the physical properties of the footwear used in the experiment. All participants provided written informed consent prior to participation. The study was conducted in accordance with the Declaration of Helsinki and was approved by the local ethics committee (project ID: 2025-31).

### 2.2 Instrumentation and experimental protocol

The experiment was conducted using a treadmill with adjustable slope settings (TR-8000 Renewal, OHTAKE-ROOT Co., Ltd., Japan). To eliminate the influence of footwear variability, all participants wore identical running shoes (Syunsoku JAPAN, Achilles, Japan). To measure plantar pressure and center of pressure (CoP), pressure measurement insoles (Pedar-X system, Novel, Germany) were inserted into the shoes. The pressure data was recorded at a sampling frequency of 100 Hz. These insoles were 2.5 mm thick and equipped with 99 sensors, each covering an area of approximately 0.391 cm^2^.

Prior to the experimental trials, participants completed a warm-up consisting of 1-minute treadmill running under each of the three slope conditions used in the main trials. Analysis of the center of pressure trajectories displayed in real-time using the Pedar software during the warm-up confirmed that all participants habitually used a rearfoot strike pattern. For each experimental condition, participants performed a 1-minute treadmill run at a constant speed of 3.33 m/s. Data were collected from a single successful trial for each condition.

The experimental conditions consisted of a combination of three slope settings—downhill running (DR; −6°), level running (LR; 0°), and uphill running (UR; +6°) (7, 11)—and two foot strike patterns: rearfoot strike (RFS) and forefoot strike (FFS), resulting in a total of six conditions. Whether participants correctly adopted the instructed foot strike pattern under each slope condition was determined based on real-time CoP trajectories displayed by the software. A trial was considered successful if the CoP at initial contact was clearly located in the heel region for RFS or forefoot region for FFS.

The order of trial conditions was randomized using a lottery method. A minimum rest period of five minutes was provided between trials to minimize the effects of fatigue.

### 2.3 Data analysis

**Figure 1:**
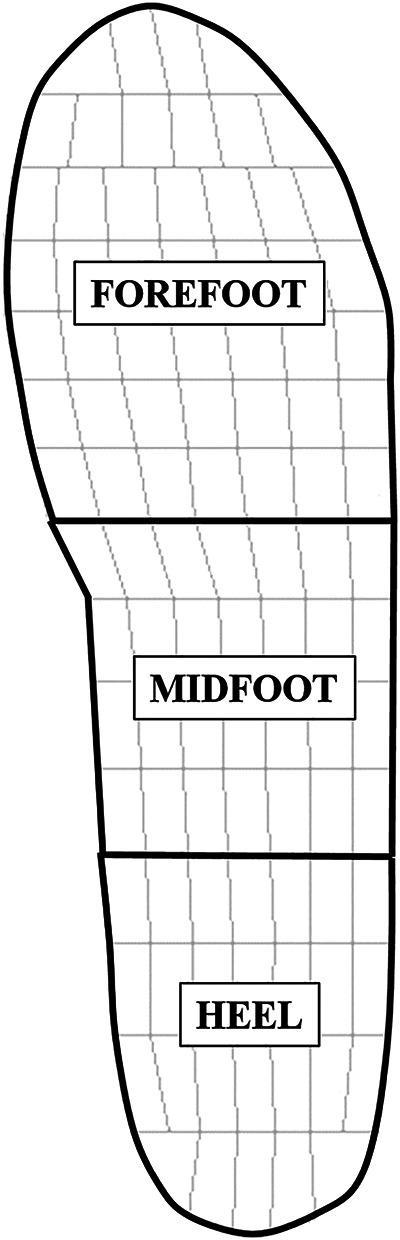
The pressure measurement insoles array is divided into 3 regions: heel, midfoot, forefoot.

**Figure 2:**
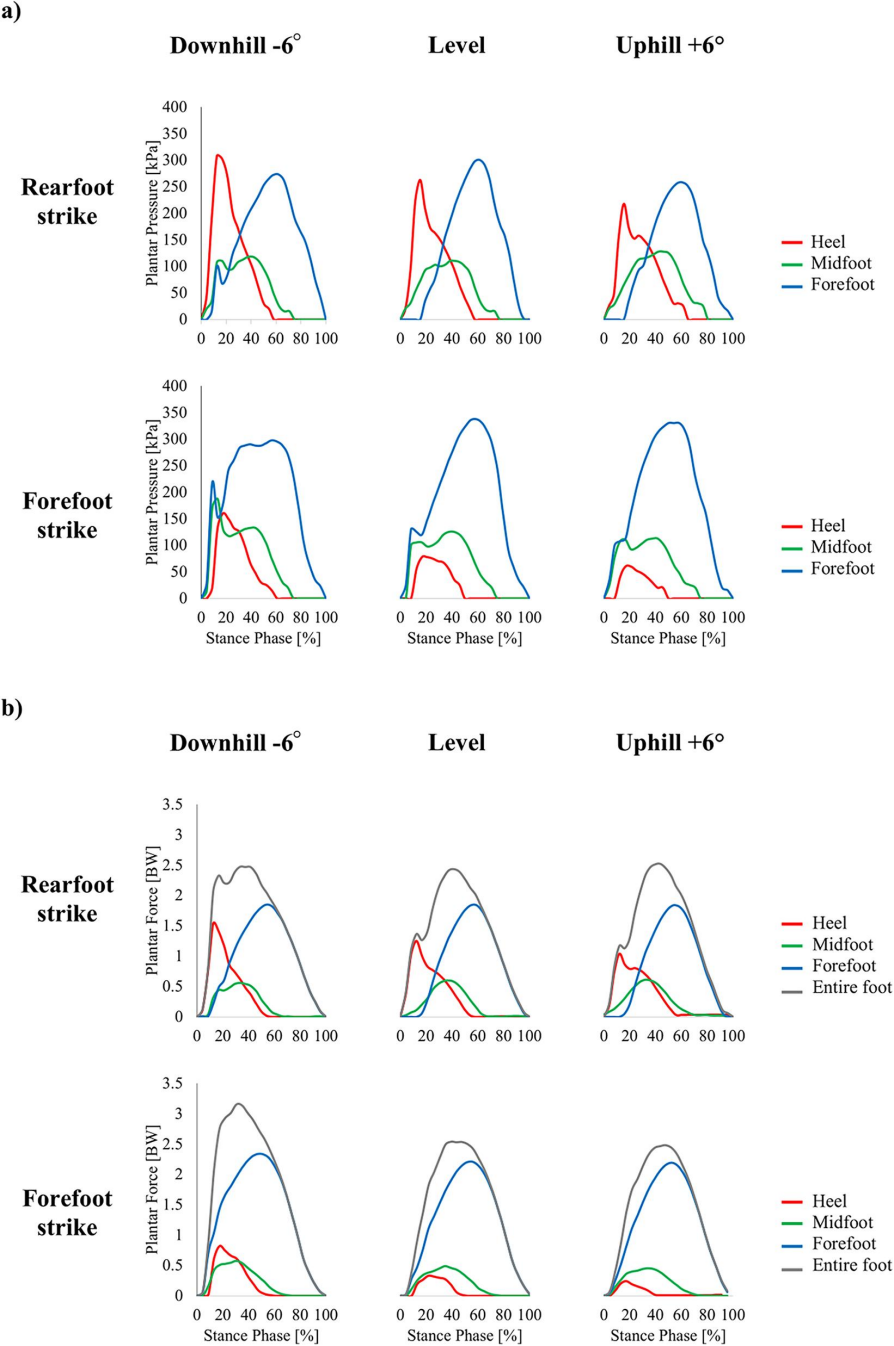
Typical waveforms of plantar pressure **(a)** and plantar force **(b)**, normalized to the percentage of the stance phase at 3.33 m/s under each running condition. Red line: heel region, green line: midfoot region, blue line: forefoot region, gray line: entire foot.

Plantar pressure was quantified using Pedar software. For each trial, 10 consecutive steps (five from each foot) were extracted from the steady-state period from 50–60 s of running at a constant speed (14). The pressure measurement insoles were divided into three anatomical regions for analysis: forefoot (40% of foot length), midfoot (30%), and heel (30%) (Figure 1) (15). From the stance phase data, the following variables were computed (Figure 2): Peak Pressure (kPa), Peak Force (16) (BW), Time to Peak Force (ms), Loading Rate (BW/s). The peak pressure and peak force were defined as the maximum values observed in each region during the stance phase. The plantar force was calculated by multiplying the pressure measured by each sensor with the sensor by its contact area. The peak force (16) was defined as the maximum vertical force calculated from plantar pressure data during the stance phase. Plantar force was derived by multiplying the pressure measured by each sensor with its contact area and then summing across all sensors. This measurement is distinct from the vertical impact peak force obtained from ground reaction force analysis, as no force plate data were collected in this study. The time to peak force was defined as the time elapsed between the onset of plantar force and the point at which the force reached its maximum value within each region. The loading rate was calculated over the initial 0%–13% of the time-normalized stance phase (17). There were two reasons for selecting this fixed early-stance window: (1) the pressure measurement insole was sampled at 100 Hz, and its sensor/conditioning characteristics reduce the temporal fidelity required to reliably identify narrow, high-frequency vertical impact peaks; and (2) a discrete vertical impact peak is often absent in forefoot-strike trials, where this approach has been used to quantify loading rate (18). Thus, a 0%–13% stance window provides a consistent method to quantify the initial force rise across strike patterns and slope conditions. For RFS conditions, the loading rate was computed in the heel region, while for FFS conditions, it was computed in the forefoot region.

In addition, spatiotemporal parameters were calculated from the insole data. The contact time for the entire foot was defined as the period between the point at which the rate of change of plantar force exceeded 1.5 kN/s at touchdown and the point at which it dropped below 1.5 kN/s at toe-off (19). The contact time for each region was defined as the duration during which plantar force was detected in that region throughout the entire contact period of the foot. Step frequency was calculated as the inverse of the step time, which was defined as the sum of contact time and flight times (i.e., the duration from toe-off of one foot to touchdown of the opposite foot). Step length was derived by dividing the treadmill belt speed by the step frequency.

### 2.4 Statistical analysis

All variables were presented as the mean ± standard deviation across all participants. Statistical analyses were conducted using JASP (version 0.18.1; JASP Team, Netherlands). To compare conditions, a two-way analysis of variance (ANOVA) was performed to compare conditions with slope (three levels) and foot strike pattern (two levels) as factors. *post hoc* tests were conducted using the Bonferroni correction. The level of statistical significance was set at *α* = 0.05. Effect sizes for ANOVA results were reported as partial eta squared (*η*²_p_) and interpreted according to Cohen's (1992) criteria: trivial (<0.01), small (0.01–0.06), medium (0.06–0.14), and large (≧0.14) (20).

## 3 Results

### 3.1 Peak pressure

Results for peak pressure are summarized in Table 1. At the heel region, significant main effects were observed in SLOPE and FSP (SLOPE: *η*²_p_ = 0.128, FSP: *η*²_p_ = 0.768), as well as an interaction between the two (*η*²_p_ = 0.028). In the RFS condition, significant differences were observed between LR and both DR and UR. Compared with LR, DR was 32.1% higher (*p* = 0.004, d = 1.497), while UR was 24.3% lower (*p* = 0.038, d = 1.133). At the forefoot region, significant main effects were observed in SLOPE and FSP (SLOPE: *η*²_p_ = 0.030, FSP: *η*²_p_ = 0.816). Regardless of the FSP, significant differences were observed between DR and LR, with DR showing lower value (*p* = 0.049, d = −0.513). Additionally, regardless of the SLOPE, a significant difference was observed between FFS and RFS, with FFS showing higher values (*p* < 0.01, d = −2.471).

**Table 1:** Variables measured by the pressure sensing insole for each regions during running at each examined conditions. Values represent means for all 11 subjects ± standard deviation.

Characteristics[unit]	Regions	Downhill (−6°)		Level (0°)		Uphill (+6°)		Main Effect of SLOPE	Main Effect of FSP	Interaction	Post hoc	
		Rearfoot Strike	Forefoot Strike	Rearfoot Strike	Forefoot Strike	Rearfoot Strike	Forefoot Strike				SLOPE	FSP
Peak Pressure (kPa)	Heel	371.0 ± 75.89	99.9 ± 43.34	280.8 ± 47.13	72.1 ± 26.63	212.5 ± 43.95	41.4 ± 18.80	**	**	**	RFS: DR > LR > UR, FFS: DR, LR > UR	
	Midfoot	170.3 ± 44.77	158.8 ± 31.93	158.0 ± 30.99	168.0 ± 47.28	152.5 ± 26.22	149.8 ± 36.27	n.s.	n.s.	n.s.	–	
	Forefoot	329.1 ± 36.75	504.7 ± 63.28	375.7 ± 44.09	524.9 ± 74.93	370.1 ± 62.13	528.2 ± 79.74	*	**	n.s.	DR < UR	RFS < FFS
Peak Force (BW)	Heel	1.45 ± 0.142	0.37 ± 0.226	1.12 ± 0.110	0.22 ± 0.123	0.86 ± 0.137	0.09 ± 0.082	**	**	**	RFS: DR > LR > UR, FFS: DR, LR > UR	
	Midfoot	0.72 ± 0.165	0.55 ± 0.115	0.72 ± 0.173	0.56 ± 0.130	0.69 ± 0.134	0.47 ± 0.113	*	**	n.s.		RFS > FFS
	Forefoot	1.65 ± 0.138	2.37 ± 0.300	1.75 ± 0.130	2.35 ± 0.227	1.75 ± 0.170	2.37 ± 0.238	n.s.	**	*	RFS: n.s., FFS: n.s.	
	Entire	2.55 ± 0.217	2.82 ± 0.361	2.55 ± 0.245	2.68 ± 0.243	2.47 ± 0.130	2.62 ± 0.270	n.s.	*	n.s.	–	RFS < FFS
Time to Peak Force (ms)	Heel	25.6 ± 4.35	42.5 ± 8.97	25.7 ± 5.41	44.5 ± 9.66	32.9 ± 14.55	60.5 ± 28.34	*	**	n.s.	DR, LR < UR	RFS < FFS
	Midfoot	76.5 ± 7.84	54.5 ± 9.39	83.7 ± 6.31	64.5 ± 5.28	86.5 ± 6.68	67.3 ± 10.80	**	**	n.s.	DR < LR, UR	RFS < FFS
	Forefoot	128.4 ± 9.73	100.0 ± 5.98	130.5 ± 9.01	107.7 ± 6.25	135.0 ± 9.75	111.0 ± 8.29	*	**	n.s.	DR < LR, UR	RFS < FFS
Loading Rate 0–13% stance phase (BW/sec)	Heel	1.36 ± 0.105		1.01 ± 0.101		0.76 ± 0.127		**	*	*	RFS: DR > LR > UR, FFS: DR > UR	
	Forefoot		1.65 ± 0.288		1.36 ± 0.239		1.30 ± 0.257					

Results of post-tests indicate significant differences. The significance level is *α* =  0.05.

DR, downhill running; LR, level running; UR, uphill running; FSP, foot strike pattern; RFS, rearfoot strike; FFS, forefoot strike.

* denotes a *p*-value less than 0.05.

** indicates a *p*-value of less than 0.001.

### 3.2 Peak force

Results for peak force are summarized in Table 1. At the heel region, significant main effects were observed in SLOPE and FSP (SLOPE: *η*²_p_ = 0.124, FSP: *η*²_p_ = 0.820) as well as an interaction between the two (*η*²_p_ = 0.016). In the RFS condition, significant differences were observed between LR and both DR and UR. Compared with LR, DR was 30.0% higher value (*p* < 0.001, d = 2.198), while UR was 23.5% lower value (*p* < 0.001, d = 1.741). At the midfoot region, significant main effects were observed in SLOPE and FSP (SLOPE: *η*²_p_ = 0.056, FSP: *η*²_p_ = 0.623). Regardless of the SLOPE, A significant difference was observed between FFS and RFS, showing a higher than FFS (*p* < 0.001, d = 1.244). At the forefoot region, a significant main effect was observed in FSP (*η*²_p_ = 0.883), as well as an interaction between the two (*η*²_p_ = 0.006). At the entire foot, a significant main effect was observed in FSP (*η*²_p_ = 0.206). Regardless of the SLOPE, a significant difference was observed between, while higher than RFS (*p* = 0.029, d = −0.695).

### 3.3 Time to peak force

Results for time to peak force are summarized in Table 1. At the heel region, significant main effects were observed in SLOPE and FSP (SLOPE: *η*²_p_ = 0.117, FSP: *η*²_p_ = 0.394). Regardless of the FSP, significant differences were observed between UR and LR, with UR showing a higher value (*p* = 0.044, d = −0.773). Additionally, regardless of the SLOPE, a significant difference was observed between FFS and RFS, with FFS showing a higher value by 75.1% (*p* < 0.001, d = −1.401). At the midfoot region, significant main effects were observed in SLOPE and FSP (SLOPE: *η*²_p_ = 0.151, FSP: *η*²_p_ = 0.649). Regardless of the FSP, significant differences were observed between DR and LR, with DR showing lower value (*p* = 0.007, d = −1.036). Additionally, regardless of the SLOPE, a significant difference was observed between FFS and RFS, with FFS showing lower value (*p* < 0.001, d = 2.418). At the forefoot region, significant main effects were observed in SLOPE and FSP (SLOPE: *η*²_p_ = 0.067, FSP: *η*²_p_ = 0.802). Regardless of the FSP, significant differences were observed between DR and LR, with DR showing lower value (*p* = 0.021, d = −0.568). Additionally, regardless of the SLOPE, a significant difference was observed between FFS and RFS, with FFS showing lower value (*p* < 0.001, d = 2.875).

### 3.4 Loading rate

Results for loading rate are summarized in Table 1. Significant main effects were observed in SLOPE and FSP (SLOPE: *η*^2^ = 0.368, FSP: *η*^2^ = 0.199), as well as a significant interaction between the two (*η*^2^ = 0.060). In the RFS condition, significant differences were observed between LR and both DR and UR. Compared with LR, DR was 32.7% higher value (*p* < 0.001, d = 1.524), while UR was 28.6% lower value (*p* < 0.001, d = 1.335). In the FFS condition, significant differences were observed between DR and LR, with DR showing higher value at 41.5% (*p* = .024, d = 1.213).

### 3.5 Spatiotemporal parameters

Results for spatiotemporal parameters are summarized in Table 2. In the step frequency, a significant main effect was observed in SLOPE (*η*²_p_ = 0.362). Regardless of the FSP, significant differences were observed between UR and LR, with UR showing higher value at 5.3% (*p* < 0.001, d = −0.768).

In the Step length, a significant main effect was observed in SLOPE (*η*²_p_ = 0.252). Regardless of the FSP, significant differences were observed between UR and DR, with UR showing lower value (*p* = 0.008, d = 0.744).

In the contact time at the heel region, a significant main effect was observed in FSP (*η*²_p_ = 0.640), as well as a significant interaction between the two (*η*²_p_ = 0.110). In the RFS condition, significant differences were observed between UR and DR, with DR showing lower value (*p* = 0.036, d = −0.859). At the midfoot region, significant main effects were observed in SLOPE and FSP (SLOPE: *η*²_p_ = 0.137, FSP: *η*²_p_ = 0.351). Regardless of the SLOPE, there was a significant difference between FFS and RFS, with RFS showing higher (*p* < 0.001, d = 1.032).

At the entire foot, a significant main effect was observed in FSP (*η*²_p_ = 0.272). Regardless of the SLOPE, there was a significant difference between FFS and RFS, with FFS 4.0% lower value (*p* < 0.001, d = 0.683).

## 4 Discussion

This study examined differences in foot strike patterns during UR, LR, and DR from the perspective of plantar pressure. Analysis of plantar pressure revealed that the waveforms of both plantar pressure and plantar force in each region varied depending on the slope conditions and foot strike patterns at the Figure 2. Hypothesis (1) was supported: plantar pressure changed according to slope differences during RFS. Specifically, peak pressure in the heel region significantly increased during downhill running. Hypothesis (2) was partially supported: peak pressure decreased during downhill running, but the expected increase during uphill running was not observed. In the case of RFS, the peak pressure on the heel region increased by 32.1% during DR compared to LR. However, it decreased by 24.3% during UR. This trend is consistent with the previous findings reported by Ho et al. (21). Furthermore, focusing on peak force in this study, it increased by approximately 30% during DR compared to LR, while it decreased by approximately 23.5% during UR. Additionally, the loading rate, an indicator of impact, increased by 32.7% during DR and decreased by 28.6% during UR. These results suggest that the braking component affects plantar loading during slope running. Previous studies have reported that the peak value of the braking component increases during DR (7, 11), which is presumed to have led to an increased peak pressure in the heel region. On the other hand, during UR, the peak value of the braking component decreases, resulting in a reduction of peak pressure in the heel region. These findings clarify the characteristics of the load placed on the feet during slope running. To prevent running-related injuries, runners can benefit from wearing highly cushioned shoes and adopting an appropriate foot strike pattern, as the impact load on the heel region increases during DR.

When focusing on pressure changes in the forefoot region, peak pressure during DR decreased by 7.4% compared to LR, exhibiting a consistent trend regardless of foot strike pattern. In contrast, no significant difference in peak pressure was observed in the forefoot region during UR. This result supports part of hypothesis (2), which predicted a decrease in peak pressure during DR, but it does not align with the expectation of an increase during UR. A possible reason for this outcome is the limited contribution of the ankle joint in generating propulsion during UR. In fact, previous studies by Roberts et al. and Yokozawa et al. have also reported that the contribution of the ankle joint is restricted during UR (22, 23). Since forefoot pressure did not increase UR, it is likely that runners adopt an alternative strategy to generate propulsion. One such strategy is an increase in step frequency. This study confirmed that step frequency increased by approximately 5% during UR compared to LR. This trend is consistent with the findings of Lussiana et al. and Padulo et al., who observed it as an adaptive strategy for UR (24, 25). Furthermore, an increase in step frequency during UR reduces vertical ground reaction force, enabling more energy-efficient running (26). Considering this, it is highly likely that runners intentionally shorten their stride length and adjust ground contact time to fine-tune the momentum needed for propulsion while maintaining speed. In other words, instead of relying on push-off through ankle plantar flexion, step frequency adjustment contributes to improving running efficiency during UR. These findings provide important insights for running training and shoe selection.

The results of this study revealed that changes in slope have a significant impact on pressure distribution in the forefoot region. Specifically, UR showed a tendency for pressure to be concentrated in localized areas of the forefoot region, whereas DR exhibited a more widespread distribution of pressure. While no significant main effect of slope was observed for peak force, a significant main effect was found for peak pressure, with peak pressure being significantly higher during UR compared to DR. This result is likely influenced by changes in contact area. During UR, the contact area in the forefoot region decreases, causing the load to concentrate in specific regions, which is presumed to lead to an increase in peak pressure. Previous studies have reported that during UR, the ankle joint angle at foot contact is more plantarflexed compared to level running, a trend that is even more pronounced in FFS. Additionally, in RFS, the degree of dorsiflexion decreases, resulting in a flatter foot strike (7). This suggests that the foot strike position shifts further forward during UR. Conversely, during DR, the foot strike position shifts backward, increasing the foot's contact area and allowing pressure to be distributed over a wider area. This adaptation is likely one of the strategies to mitigate impact forces upon landing. Furthermore, it was found that the time to peak in the forefoot region was longer during UR compared to DR. This result suggests that the forward movement of the center of pressure is delayed, causing the midfoot region to lift off the ground before peak pressure occurs. As a result, the contact area in the forefoot decreases even further, leading to a more localized concentration of pressure. Overall, these findings suggest that slope influences plantar pressure distribution, fundamentally altering the way load is applied during running. In particular, UR leads to increased pressure due to a reduction in contact area, whereas DR results in pressure dispersion due to an increase in contact area. These insights are crucial for understanding load characteristics and adaptive strategies in running and should be considered in both running form adjustments and shoe selection.

The results of this study suggested that appropriately adjusting foot strike patterns during UR and DR can effectively reduce lower limb load. In DR, it was found that a FFS contributes to impact absorption. This is because FFS results in lower peak pressure, peak force, and loading rate in the heel region compared to RFS. Excessive impact load on the heel region has been reported to be associated with an increased risk of patellofemoral pain and plantar fasciitis (27, 28). Therefore, choosing FFS during DR may be an effective strategy for preventing running-related injuries. On the other hand, during UR, RFS may help reduce the burden on the triceps surae. In this study, RFS was found to have lower peak pressure and peak force in the forefoot region. This suggests that the plantar flexion torque at push-off is smaller, thereby reducing the load on the triceps surae. Additionally, in LR, RFS has been reported to limit the elongation of the triceps surae at foot contact, resulting in less negative work on the achilles tendon compared to FFS (29). Moreover, previous research has shown that as the slope increases during UR, the mechanical energy of the achilles tendon also increases (30). This implies that choosing FFS could place even greater stress on the achilles tendon. Therefore, selecting RFS during UR is a reasonable strategy to minimize excessive strain on both the triceps surae and the achilles tendon. However, on steep inclines, the ankle's range of motion constraints may make it difficult to maintain an RFS, forcing runners to adopt an FFS instead. In such cases, adjusting stride length and strengthening the relevant muscles may be necessary to mitigate the excessive load associated with FFS. Based on these findings, selecting an appropriate foot strike pattern according to the slope running is crucial for both injury prevention and performance improvement. Future studies should explore more specific adaptive strategies that consider individual runner characteristics and training conditions.

In this study, it was observed that the plantar pressure waveform in the forefoot region during FFS exhibited a bimodal pattern in some participants at the Figure 2A. Additionally, the loading rate tended to decrease in the order of DR, LR, and UR. These results suggest that changes in load transfer and foot contact position during landing may be involved. Specifically, the gradual transfer of load across different areas of the forefoot likely resulted in the observed bimodal pressure distribution. Furthermore, differences in impact absorption mechanisms due to slope changes may have contributed to the variations in loading rate. In particular, while impact forces increase during DR, they are mitigated in UR through adjustments in ankle angle and muscle activity. The significance of this study lies in quantitatively clarifying the impact of slope changes on plantar pressure distribution in FFS. By utilizing pressure measurement insoles, these phenomena could be visualized and quantitatively evaluated. Previous studies on impact forces in FFS have been limited, but this study demonstrated that plantar pressure analysis is an effective method for quantifying foot strike impact in FFS (31, 32). These findings have potential applications in assessing the risk of running-related injuries and guiding appropriate shoe design. Furthermore, as fundamental data demonstrates the quantitative impact of slope and foot strike patterns on lower limb load, this research can contribute to optimizing running form and training guidance. Future studies should further explore adaptive strategies while considering individual runner characteristics.

This study has four limitations related to the measurement methodology. First, the measurements in this study were conducted using a pressure measurement insole with a sampling frequency of 100 Hz. This frequency is lower than that of force plates, which typically operate at 1,000 Hz or higher (1, 19, 33, 34), and thus imposes limitations on capturing rapid load changes immediately after foot contact with high precision. However, by employing a pressure measurement insole, this study was able to evaluate the effects of slope and foot strike patterns on plantar pressure. Notably, force plates are challenging to use in outdoor or inclined environments, making the method used in this study advantageous for measurements under practical conditions. Second, while the pressure measurement insole used in this study can measure vertical forces, it cannot assess braking or propulsive forces. Slope has been suggested to have a significant influence on propulsion force during running (7, 11), and accurately capturing this element is crucial for understanding running mechanics. Future research should incorporate measurement methods capable of capturing horizontal forces to enable a more detailed analysis of propulsion and braking forces. This would lead to a more comprehensive understanding of the propulsion mechanisms in UR and DR, with potential applications in training guidance and shoe design. Third, although slope conditions caused minor fluctuations in the timing of the heel peak force during RFS, all peaks occurred early in stance. Thus, these variations are unlikely to substantially affect the validity of our loading rate calculation. Finaly, we acknowledge that including only male participants is a limitation that restricts the generalizability of our findings to female runners. Previous research has reported sex differences in running biomechanics, including variations in foot morphology, stride patterns, and ground contact characteristics (35), which may directly influence plantar pressure distribution. Thus, distinct plantar loading patterns may exist between sexes that our study could not address. While the current sample size was appropriate for this exploratory investigation of foot strike effects during slope running, future studies should recruit both male and female participants in sufficient numbers to allow sex-stratified analyses and to examine sex as a potential moderating factor in plantar pressure outcomes.

## 5 Conclusion

This study examined the effects of different foot strike patterns on plantar pressure during slope running. In downhill running, forefoot strike reduced peak pressure at the heel region compared to level running, while rearfoot strike increased it. In uphill running, forefoot strike showed no change in peak pressure at the forefoot region, while rear foot strike reduced it compared to level running. These findings suggest that selecting the appropriate foot strike pattern, forefoot strike for downhill and rearfoot strike for uphill running can help reduce lower limb load and improve performance. These findings establish the biomechanical foundation necessary for developing evidence-based training interventions and strike pattern modification protocols in slope running contexts.

## Data Availability

The datasets presented in this article are not readily available because Unauthorized reproduction prohibited. Requests to access the datasets should be directed to Toshio Yanagiya, tyanagi@juntendo.ac.jp.
